# Factors associated with artificial airway retention after skull base chordoma resection: A retrospective cohort study

**DOI:** 10.3389/fneur.2022.992308

**Published:** 2022-09-09

**Authors:** Yuxuan Fu, Yun Yu, Yidan Cui, Jing Wang, Bo Ma, Minyu Jian, Jingxin Yao, Longnian Jing, Jiwei Bai, Ruquan Han

**Affiliations:** ^1^Department of Anesthesiology, Beijing Tiantan Hospital, Capital Medical University, Beijing, China; ^2^Department of Neurosurgery, Beijing Tiantan Hospital, Capital Medical University, Beijing, China

**Keywords:** chordoma, artificial airway, postoperative pulmonary complications, anesthesiology, perioperative

## Abstract

**Background:**

Chordoma is a malignant bone and soft tissue tumor derived from embryonic notochord remnants, and skull base chordoma accounts for ~1/3 of all chordoma cases. Skull base chordoma is closely related to the brainstem and cranial nerves and has a high recurrence rate. The purpose of this study was to investigate the influence of the timing of tracheal extubation on perioperative pulmonary complications. We also aimed to explore predictors of postoperative artificial airway (AA) retention in patients with skull base chordoma.

**Methods:**

This was a single-center, retrospective cohort study. The study population included all skull base chordoma patients undergoing surgical treatment between January 2019 and December 2021 at Beijing Tiantan Hospital. The primary outcome was the incidence of postoperative pulmonary complications. Several patient characteristics were evaluated for potential associations with AA retention.

**Results:**

A total of 310 patients with skull base chordoma were enrolled. The frequency of AA retention after surgery for skull base chordoma was 30.97%. The incidence of postoperative pulmonary complications was much lower in those without AA retention (3.74 vs. 39.58%, *P* < 0.001). Factors with the highest point estimates for the odds of AA retention included body mass index, cranial nerve involvement, maximum tumor diameter, operative method, hemorrhage volume, operative duration and intraoperative mechanical ventilation duration.

**Conclusions:**

In this retrospective cohort study, most of the factors associated with postoperative airway retention were closely related to the patient's tumor characteristics. These data demonstrate that respiratory management in patients with skull base chordoma remains an ongoing concern.

## Introduction

Chordoma is a rare malignant bone and soft tissue tumor derived from embryonic notochord remnants. The incidence of chordoma has been reported as 0.08 per 100,000, with skull base chordoma accounting for ~1/3 of all chordoma cases (1, 2). Skull base chordoma is closely related to the brainstem and cranial nerves and has a high recurrence rate. The 5-year survival rate is only 60–70% (3). Chordoma is not sensitive to conventional low-dose radiation, and high-dose radiation may damage surrounding brain tissue and nerves (2, 4). Treatment of chordoma has been based on surgical resection and radiotherapy. A growing body of evidence to support the particles-ion radiotherapy is an effective treatment.

The growth rate of skull base chordoma tumors is slow, and the clinical manifestations are mainly mass effect and compression symptoms. Compression of cranial nerves by the tumor can result in preoperative neurological dysfunction, with clinical manifestations including dysphagia, choking on water, and decreased pharyngeal reflex. There are 9–12 pairs of posterior cranial nerves, including the glossopharyngeal nerve, vagus nerve, accessory nerve, and hypoglossal nerve. La Corte et al. conducted a retrospective study of 59 skull base chordoma patients and found that the preoperative clinical symptoms, tumor resection degree, surgeon's experience, and postoperative complications were significant factors affecting the prognosis (5). Additionally, long-term artificial airway (AA) retention may lead to pulmonary complications such as respiratory infection. However, in the case of damage to the posterior cranial nerves, early active extubation can lead to aspiration pneumonia due to the weakened protective airway reflex and increase the incidence of perioperative pulmonary complications. Therefore, choosing the appropriate endotracheal extubation time is particularly important. To date, there have been no studies on predictors of postoperative AA retention in patients with skull base chordoma.

This study aimed to explore predictors of postoperative AA retention in patients with skull base chordoma using retrospective observational data from Beijing Tiantan Hospital. We also aimed to investigate the influence of the timing of tracheal extubation on perioperative pulmonary complications.

## Materials and methods

This was a single-center, retrospective cohort study conducted at Beijing Tiantan Hospital. The trial protocol was approved by the Clinical Research Ethics Committee of Beijing Tiantan Hospital (approval no. KY2021-112-02), and the requirement for informed consent was waived.

### Participants

The study population included all skull base chordoma patients undergoing surgical treatment between January 2019 and December 2021 at Beijing Tiantan Hospital. Patients younger than 18 years of age and patients without anesthesia records were excluded.

### Data collection and outcome assessment

All patients underwent standardized preoperative preparation according to our hospital. Anticoagulants and antiplatelet drugs were discontinued for a specified period of time and heparin bridging therapy was performed as needed. Baseline data included demographic characteristics, preoperative comorbidities, smoking and alcohol history, and laboratory test results. Intraoperative data included the types and doses of anesthetics/medications, vital signs, fluid balance and blood transfusions, intraoperative mechanical ventilation duration, and operative duration. In addition, unplanned secondary endotracheal intubation and unplanned secondary operations were recorded. The patients were divided into two groups according to whether the AA was retained postoperatively: patients with AA retention (AA group) and without AA retention (WOAA group). AAs included those present after oral endotracheal intubation, nasal endotracheal intubation, and tracheotomy. Tumor diameter was measured by a senior professional radiologist based on enhanced magnetic resonance imaging of the head.

The primary outcome was the incidence of postoperative pulmonary complications. The definition of perioperative pulmonary complications included respiratory infection, respiratory failure, pleural effusion, atelectasis, pneumothorax, bronchospasm, and aspiration pneumonia. The Clavien–Dindo classification was used to categorize pulmonary lesions. Complications of grade II or above were used to calculate the incidence rate (6). Secondary outcomes included the incidence of extrapulmonary complications, postoperative length of hospital stay, postoperative length of intensive care unit (ICU) stay, and rate of unplanned secondary endotracheal intubation. Postoperative extrapulmonary complications were defined as new events outside the respiratory system, including neurovascular complications (stroke, transient ischemic accident), cardiovascular complications (acute coronary syndrome, circulatory insufficiency, congestive heart failure, new-onset arrhythmia), thromboembolic complications (pulmonary embolism, deep venous thrombosis), gastrointestinal complications (gastrointestinal hemorrhage, acute pancreatitis, ileus), surgical complications (surgical-site infection, surgical bleeding), infectious complications (sepsis, septic shock), and liver and kidney complications (acute hepatic injury, acute kidney injury) (6). The diagnosis of all postoperative complications was confirmed by two senior specialists. Surgical site infection is defined as infection related to an operative procedure, occurred at or near the surgical incision within 30 days of the procedure or cerebrospinal fluid tests confirm an intracranial infection (6). A poor prognosis was defined as death during hospitalization or Glasgow score of eight or less at discharge.

### Statistical analysis

EmpowerStats software and R software (R version 4.2.0) were used for statistical analysis. The Kolmogorov–Smirnov test was applied for continuous variables with a normal distribution. Data conforming to a normal distribution are represented as the mean ± standard deviation (SD). Nonnormally distributed data are expressed as medians and interquartile ranges. The independent *T*-test or Mann–Whitney U test was performed according to the data distribution. Classification variables are expressed as a percentage and were analyzed using the chi-square test or Fisher's exact test. Based on the univariate logistic regression analysis, possible factors influencing postoperative pulmonary complications were found, and variables with a *P* < 0.05 were included in the multiple regression analysis. By logistic regression, odds ratios (ORs) and 95% confidence intervals (CIs) were calculated to evaluate the factors associated with postoperative AA retention. According to the multivariate analysis, continuous variables related to postoperative AA retention were selected to draw receiver operating characteristic (ROC) curves. *P* < 0.05 was considered statistically significant.

## Results

**Figure 1:**
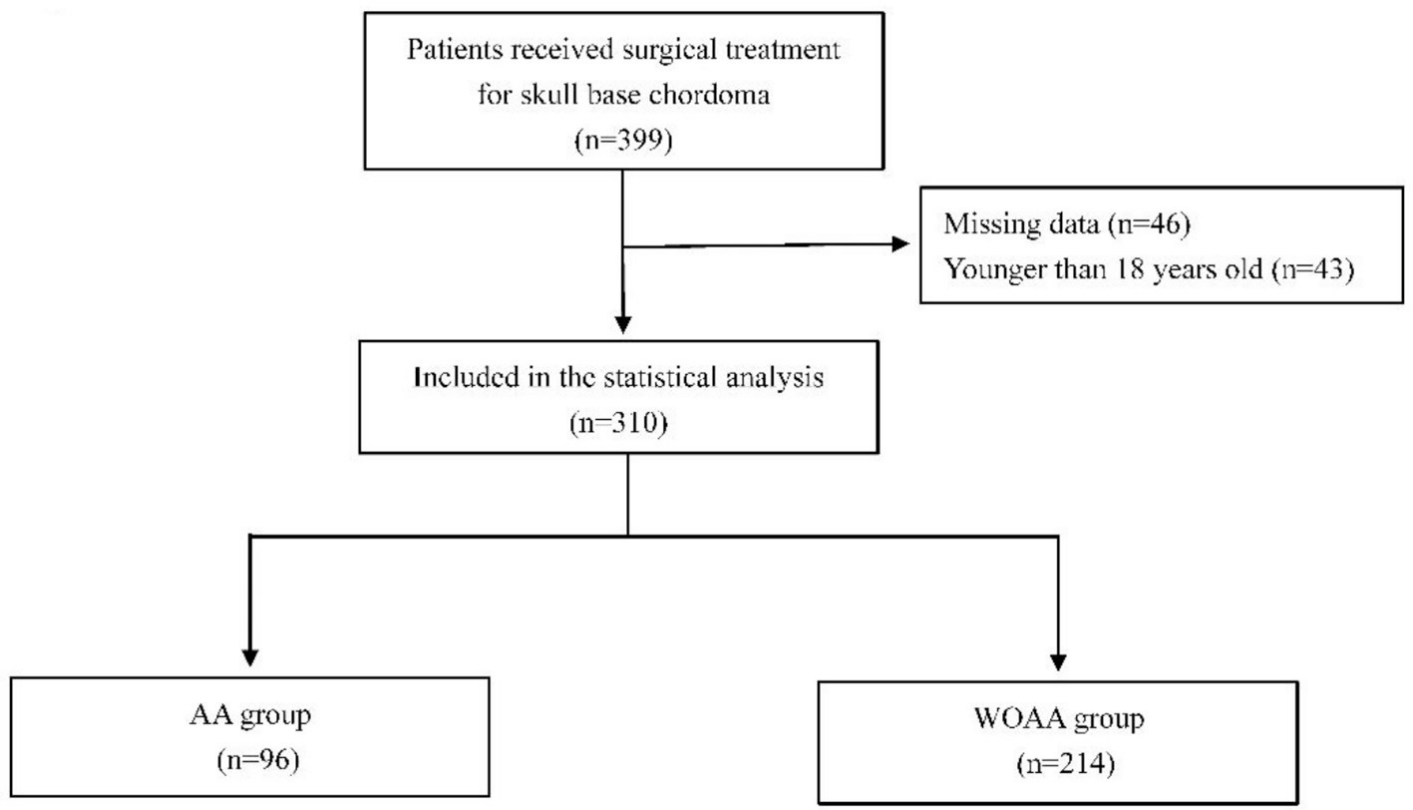
Patient flow chart.

From January 2019 to December 2021, 399 patients underwent surgical treatment for skull base chordoma at Beijing Tiantan Hospital. Among them, 43 patients were younger than 18 years, and anesthesia data were unavailable for 46 patients. Thus, a total of 310 patients were included in the statistical analysis (Figure 1).

### Patient characteristics

The baseline characteristics of the patients in the two groups are shown in Table 1. There were no significant differences between the two groups in terms of smoking history, diabetes mellitus, hypertension, stroke history, heart disease, respiratory disease, recurrence, or maintenance of anesthesia. The operative duration, intraoperative mechanical ventilation duration, maximum tumor diameter, and hemorrhage volume were significantly greater in those with than those without AA retention. In addition, there were significant differences in age, body mass index (BMI), sex, posterior cranial nerve involvement, ASA physical status and the operative method between the two groups.

**Table 1:** Patient characteristics.

	**Total**	**WOAA group**	**AA group**	***P-value***
No. of cases	310	214 (69.03%)	96 (30.97%)	
Age (yr)	50.0 (37.0–59.0)	52.0 (39.8–60.0)	48.0 (34.0–56.0)	0.018
BMI (kg/m^2^)	24.1 ± 3.7	24.6 ± 3.5	23.2 ± 4.0	0.002
Sex				<0.001
Male	186 (60.00%)	142 (66.36%)	44 (45.83%)	
Female	124 (40.00%)	72 (33.64%)	52 (54.17%)	
Smoking history	42 (13.55%)	31 (14.49%)	11 (11.46%)	0.471
Diabetes mellitus	12 (3.87%)	10 (4.67%)	2 (2.08%)	0.274
Hypertension	44 (14.19%)	35 (16.36%)	9 (9.38%)	0.103
Stroke	4 (1.29%)	3 (1.40%)	1 (1.04%)	0.795
Heart disease	9 (2.90%)	7 (3.27%)	2 (2.08%)	0.565
Respiratory disease	4 (1.29%)	4 (1.87%)	0 (0.00%)	0.178
Relapse	170 (54.84%)	115 (53.74%)	55 (57.29%)	0.561
Cranial nerve involvement	59 (19.03%)	17 (7.94%)	42 (43.75%)	<0.001
ASA physical status	310			0.008
I	18 (5.8%)	13 (6.1%)	5 (5.2%)	
II	255 (82.3%)	184 (86.0%)	71 (74.0%)	
III	35 (11.3%)	17 (7.9%)	18 (18.8%)	
IV	1 (0.3%)	0 (0%)	1 (1.0%)	
V	1 (0.3%)	0 (0%)	1 (1.0%)	
Operative method				<0.001
Endoscopy	230 (74.19%)	184 (85.98%)	46 (47.92%)	
Craniotomy	80 (25.81%)	30 (14.02%)	50 (52.08%)	
Maintenance of anesthesia				0.169
TIVA	18 (5.81%)	15 (7.01%)	3 (3.12%)	
Combined intravenous–inhalation anesthesia	279 (90.00%)	188 (87.85%)	91 (94.79%)	
Inhalation	13 (4.19%)	11 (5.14%)	2 (2.08%)	
Tranexamic acid	17 (5.48%)	10 (4.67%)	7 (7.29%)	0.349
Maximum tumor diameter (mm)	40.0 (32.0–50.0)	38.0 (30.0–48.5)	45.0 (37.0–58.0)	<0.001
Hemorrhage volume (ml)	400.00 (200.00–800.00)	300.00 (200.00–600.00)	600.00 (300.00–1000.00)	<0.001
Hemorrhage volume (ml/kg)	5.76 (3.33–11.67)	4.76 (2.87–8.60)	10.00 (4.98–17.94)	<0.001
Operative duration (min)	235.00 (160.00–340.00)	194.00 (140.00–255.00)	375.00 (300.00–466.25)	<0.001
Intraoperative mechanical ventilation duration (min)	287.50 (210.00–418.75)	250.00 (185.75–315.00)	447.50 (363.75–547.50)	<0.001

AA, with a retained artificial airway; BMI, body mass index; TIVA, total intravenous anesthesia; WOAA, without a retained artificial airway; ASA, American Society of Anesthesiologists.

### Postoperative outcomes

In this study, we found that the rate of AA retention after surgery for skull base chordoma was 30.97%; buccal endotracheal intubation was maintained in 55 patients, nasal endotracheal intubation in 22 patients, and tracheotomy in 19 patients.

The outcomes of the patients are shown in Table 2. In this study, 46 patients developed perioperative pulmonary complications, including 38 patients with AA retention and eight without AA retention. The incidence of postoperative pulmonary complications was much lower in those without AA retention (3.74 vs. 39.58%, *P* < 0.001). The incidence of postoperative extrapulmonary complications was 32.29 and 19.63% in those with and without AA retention, respectively (*P* = 0.015). Both the length of postoperative hospital stay and the length of ICU stay were extended in the AA group (*P* < 0.001). There were two patients (0.93%) with an unfavorable prognosis in the WOAA group and 23 patients (23.96%) in the AA group (*P* < 0.001).

**Table 2:** Outcome variables, stratified by AA retention.

	**Total**	**WOAA group**	**AA group**	***P-value***
No. of cases	310	214	96	
Pulmonary complication	46 (14.84%)	8 (3.74%)	38 (39.58%)	<0.001
Respiratory infection	40 (12.90%)	7 (3.27%)	33 (34.38%)	<0.001
Extrapulmonary complications	73 (23.55%)	42 (19.63%)	31 (32.29%)	0.015
Unplanned secondary endotracheal intubation	17 (5.48%)	11 (5.14%)	6 (6.25%)	0.691
Unplanned secondary operation	16 (5.16%)	9 (4.21%)	7 (7.29%)	0.256
Postoperative hospital stay (day)	8.00 (6.00–13.00)	7.00 (5.00–9.00)	12.00 (8.00–16.00)	<0.001
Postoperative ICU stay (day)	0.00 (0.00–1.00)	0.00 (0.00–1.00)	1.00 (1.00–4.25)	<0.001
Discharged				<0.001
Home	285 (91.94%)	212 (99.07%)	73 (76.04%)	
Rehabilitation hospital	21 (6.77%)	2 (0.93%)	19 (19.79%)	
Death	4 (1.29%)	0 (0.00%)	4 (4.17%)	
Poor prognosis	25 (8.06%)	2 (0.93%)	23 (23.96%)	<0.001

AA, with a retained artificial airway; ICU, intensive care unit; WOAA, without a retained artificial airway.

There was no significant difference between the two groups in terms of unplanned secondary endotracheal intubation and unplanned secondary surgery. Six patients in the AA group underwent secondary endotracheal intubation; causes of secondary endotracheal intubation included dyspnea, poor cough reflex, and respiratory infection. Eleven patients in the WOAA group underwent secondary endotracheal intubation; reasons for endotracheal intubation included the need for a second operation, postoperative local cerebral edema, reflux aspiration, and pulmonary infection. Of the 17 patients who underwent secondary endotracheal intubation, one died, and seven had a poor prognosis and required continued treatment in the rehabilitation hospital.

The artificial airway was retained for 20 h (15–219 h) after operation. The patients who retained the artificial airway after operation were divided into two groups: those who retained the artificial airway for <24 h after operation were in the short-term group, and the others were in the long-term group. There were 50 people in the short-term group and 46 people in the long-term group. Short-term group of postoperative artificial airway retention time is 15.5 h (13.0–17.0 h), long-term group is 230.0 h (144.8–321.0 h).

### Predictors associated with postoperative AA retention

Univariate analysis showed that women were more likely than men to require AA retention postoperatively (OR = 2.33, 95% CI: 1.43–3.81; *P* = 0.0007). Patients with cranial nerve involvement were also more likely to require AA retention postoperatively (OR = 9.01, 95% CI: 4.76–17.07; *P* < 0.0001). Compared with endoscopic surgery, patients who underwent craniotomy had a higher risk of postoperative AA retention (OR = 6.67, 95% CI: 3.82–11.63; *P* < 0.0001) (Table 3). In addition, the maximum tumor diameter, intraoperative hemorrhage volume, operative duration, and intraoperative mechanical ventilation duration were related to postoperative AA retention.

**Table 3:** Univariate analysis for risk factors associated with retained artificial airways.

	**Statistics**	**Retained artificial airways**	***P*-value**
Sex			0.0007
Male	186 (60.00%)	1.0	
Female	124 (40.00%)	2.33 (1.43, 3.81)	
Age (yr)	48.3 ± 13.7	0.98 (0.96, 1.00)	0.0155
Relapse	170 (54.84%)	1.15 (0.71, 1.88)	0.5612
Smoking history	42 (13.55%)	0.76 (0.37, 1.59)	0.4724
Cranial nerve involvement	59 (19.03%)	9.01 (4.76, 17.07)	<0.0001
BMI (kg/m^2^)	24.1 ± 3.7	0.90 (0.84, 0.96)	0.0024
Maximum tumor diameter (mm)	42.22 ± 14.19	1.04 (1.02, 1.07)	<0.0001
Hemorrhage volume (ml)	400.0 (200.0–800.0)	1.00 (1.00, 1.00)	0.0001
Hemorrhage volume (ml/kg)	5.76 (3.33–11.67)	1.07 (1.04, 1.09)	<0.0001
Operative duration (min)	235.0 (160.0–340.0)	1.01 (1.01, 1.02)	<0.0001
Intraoperative mechanical ventilation duration (min)	287.50 (210.00–418.75)	1.01 (1.01, 1.02)	<0.0001
Maintenance of anesthesia			
TIVA	18 (5.81%)	1.0	
Combined intravenous–inhalation anesthesia	279 (90.00%)	2.42 (0.68, 8.57)	0.1707
Inhalation	13 (4.19%)	0.91 (0.13, 6.40)	0.9237
Operative method			
Endoscopy	230 (74.19%)	1.0	
Craniotomy	80 (25.81%)	6.67 (3.82, 11.63)	<0.0001

BMI, body mass index; TIVA, total intravenous anesthesia.

Two multifactor analysis models were established (Table 4). Model one was adjusted for sex and age, while model two was adjusted for sex, age, and smoking history. After adjusting for potential confounding factors, the multivariate analysis showed that BMI (OR = 0.91, 95% CI: 0.85–0.97; *P* = 0.0077), cranial nerve involvement (OR = 10.12, 95% CI: 5.11–20.05; *P* < 0.0001), maximum tumor diameter (OR = 1.05, 95% CI: 1.03–1.07; *P* < 0.0001), operative method (OR = 6.85, 95% CI: 3.84–12.21; *P* < 0.0001), hemorrhage volume (OR = 1.06, 95% CI: 1.03–1.09; *P* < 0.0001), operative duration (OR = 1.01, 95% CI: 1.01–1.02; *P* < 0.0001), and intraoperative mechanical ventilation duration (OR = 1.01, 95% CI: 1.01–1.02; *P* < 0.0001) were all associated with postoperative AA retention.

**Table 4:** Multifactorial regression for risk factors associated with retained artificial airways.

**Exposure**	**Adjust I**	***P*-value**	**Adjust II**	***P*-value**
Relapse	1.19 (0.72, 1.96)	0.5073	1.18 (0.71, 1.96)	0.5133
Cranial nerve involvement	9.58 (4.90, 18.73)	<0.0001	10.12 (5.11, 20.05)	<0.0001
Maximum tumor diameter (mm)	1.05 (1.02, 1.07)	<0.0001	1.05 (1.03, 1.07)	<0.0001
Operative method				
Endoscopy	1.0		1.0	
Craniotomy	6.85 (3.84, 12.23)	<0.0001	6.83 (3.83, 12.21)	<0.0001
Maintenance of anesthesia				
TIVA	1.0		1.0	
Combined intravenous–inhalation anesthesia	2.25 (0.62, 8.15)	0.2169	2.30 (0.63, 8.32)	0.2055
Inhalation	0.99 (0.14, 7.22)	0.9913	1.03 (0.14, 7.56)	0.9766
Hemorrhage volume (ml)	1.00 (1.00, 1.00)	0.0001	1.00 (1.00, 1.00)	0.0001
Hemorrhage volume (ml/kg)	1.06 (1.03, 1.09)	<0.0001	1.06 (1.03, 1.09)	<0.0001
Operative duration (min)	1.01 (1.01, 1.02)	<0.0001	1.01 (1.01, 1.02)	<0.0001
Intraoperative mechanical ventilation duration (min)	1.01 (1.01, 1.02)	<0.0001	1.01 (1.01, 1.02)	<0.0001
BMI (kg/m^2^)	0.91 (0.85, 0.98)	0.0083	0.91 (0.85, 0.97)	0.0077

Adjust I model adjust for: sex; age.

Adjust II model adjust for: sex; age; smoke.

BMI, body mass index; TIVA, total intravenous anesthesia.

**Figure 2:**
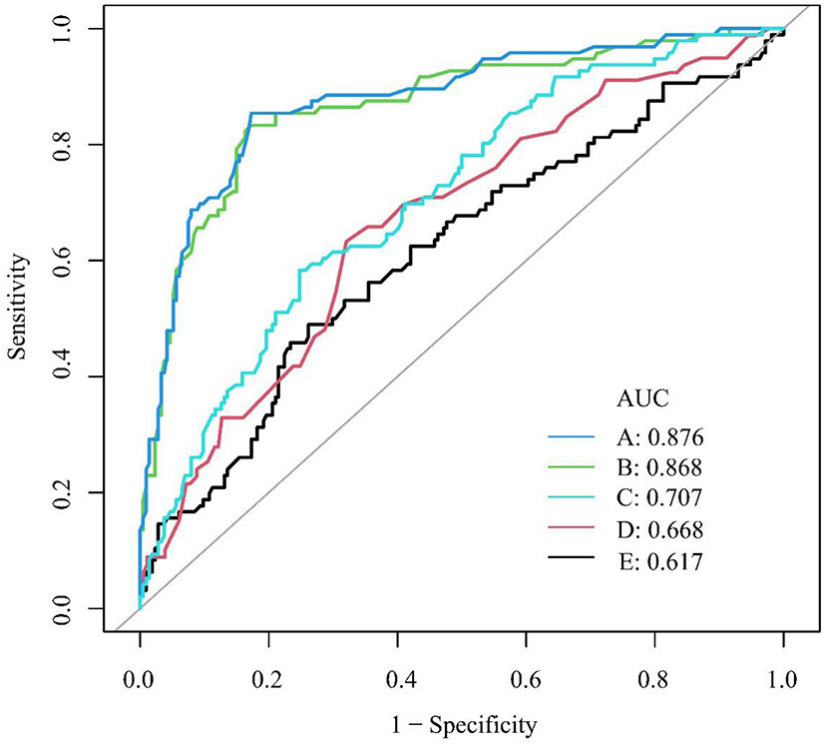
ROC curve for AA retention. **(A)** Duration of intraoperative mechanical ventilation (min); **(B)** operative duration (min); **(C)** hemorrhage volume (ml/kg); **(D)** maximum tumor diameter (mm); **(E)** BMI (kg/m^2^). AUC, area under the ROC curve; BMI, body mass index; ROC, receiver operating characteristic.

According to the multivariate analysis, continuous variables related to AA retention were selected for ROC curve analysis, and the results are shown in Figure 2. The area under the ROC curve (AUC) of the BMI was 0.61 (95% CI: 0.54–0.67). When the optimal threshold was 22.64, the specificity was 0.74, and the sensitivity was 0.49. The AUC of the maximum tumor diameter was 0.66 (95% CI: 0.59–0.73), with a specificity and sensitivity of 0.68 and 0.63, respectively, when the optimal threshold was 43.50 mm. The AUC of the operative duration was 0.85 (95% CI: 0.81–0.89), with a specificity and sensitivity of 0.83 and 0.83, respectively, when the optimal threshold was 282.50 min. The AUC of the intraoperative mechanical ventilation duration was 0.86 (95% CI: 0.82–0.91), with a specificity and sensitivity of 0.83 and 0.85, respectively, when the optimal threshold was 343.50 min. The AUC of the hemorrhage volume was 0.71 (95% CI: 0.64–0.77), when the optimal cutoff point was 8.63 ml/kg, with a specificity of 0.75 and sensitivity of 0.58.

## Discussion

Chordoma is a rare malignant and aggressive tumor with a poor prognosis. Skull base chordomas are often more complex than other chordomas (7, 8). This study retrospectively analyzed the incidence of postoperative pulmonary complications in patients with skull base chordoma and explored the risk factors for postoperative AA retention. Among them, 30.97% of patients required postoperative AA retention, which was found to be associated with an increased frequency of pulmonary complications and a poor prognosis. The results of this study are consistent with those of studies of nonneurosurgical patients (9–12). Prolonged AA retention can lead to complications, including lung infection, glottis narrowing, and difficulty swallowing (13). Postoperative pulmonary complications are also associated with an increased mortality rate, length of hospital stay, and cost of hospitalization (14–19).

In neurosurgical patients, prolonged surgery, substantial hemorrhage, diabetes mellitus, chronic obstructive pulmonary disease, preoperative leukocytosis, ASA≥3 classification, and skull base occupation are risk factors for postoperative pneumonia (20). Additionally, symptoms of cranial nerve involvement, prolonged surgery, long-term mechanical ventilation, decreased postoperative consciousness, and skull base surgery increase the incidence of pulmonary complications in neurosurgical patients during the perioperative period (21, 22). Furthermore, postoperative blood transfusions, posterior cranial nerve compression, prolonged ICU stay, and tracheotomy are independent predictors of postoperative pulmonary complications in skull base surgery (20). Furthermore, postoperative pulmonary complications are significantly associated with a poorer prognosis, including higher rates of secondary surgery, readmission, mortality, and extended hospitalization, after craniocerebral tumor surgery (20).

Retained AAs and mechanical ventilation can provide direct access to the lungs for pathogens and reduce the immune barrier function of the mucosa, which may increase the risk of postoperative pulmonary complications (10). We identified several risk factors for perioperative AA retention, and the possibility of AA retention is considerable for patients with specific characteristics. Posterior cranial nerve involvement, craniotomy for chordoma resection, BMI > 22.64 kg/m^2^, maximum tumor diameter > 43.5 mm, operative duration > 282.50 min, intraoperative mechanical ventilation duration > 343.50 min, and hemorrhage volume > 8.63 ml/kg were all independent risk factors for postoperative AA retention. Considering that most factors related to AA retention are tumor characteristics, few factors can be actively addressed. Therefore, clinical work may focus on reducing the incidence of pulmonary complications in high-risk patients who require AA retention. A meta-analysis of 178 randomized controlled studies found that exercise interventions and inspiratory muscle training reduced perioperative pulmonary complications in patients undergoing elective major surgery (23). Laurent et al. found that preoperative ventilator endurance training could improve respiratory muscle endurance and reduce the incidence of postoperative pulmonary complications (24).

More than 20% of complications in respiratory management occur during extubation, with severe consequences, including hypoxia and death (13). Although many guidelines focus on endotracheal intubation, there is limited literature on endotracheal extubation at the end of surgery. At the end of the surgery, endotracheal extubation or AA retention is necessary according to the patient's specific situation. In patients with skull base chordoma, cranial nerve involvement results in impaired cranial nerve function and poor protective airway reflexes. Therefore, caution is needed when considering whether to retain AAs. In addition, surgeons should minimize the operative duration and hemorrhage volume, and anesthetists should refine perioperative airway management methods. There are some limitations to this study. First, this was a single-center, retrospective study, and we could only determine the patient's condition through medical records. In addition, we could not collect intraoperative mechanical ventilation parameters, including the tidal volume, respiratory rate, airway pressure, and other data. Previous randomized controlled studies have found no significant difference in the incidence of pulmonary complications 7 days after surgery between adults patients ventilated with a low intraoperative tidal volume and those ventilated with a conventional tidal volume (25). There were significant differences in maximum tumor diameter, hemorrhage volume, operative duration, and intraoperative mechanical ventilation duration between the two groups. The above factors will affect the postoperative status of patients, making patients more sensitive to infection and other complications. It is difficult to draw a firm line between these factors as direct causes of poor prognosis or as reasons for the preservation of the artificial airway after surgery. However, the results of this study need to be validated by large prospective studies.

## Conclusion

Postoperative AA retention in patients with skull base chordoma is associated with an increased incidence of pulmonary complications in the perioperative period. BMI, tumor compression of posterior cranial nerves, maximum tumor diameter, operative method, intraoperative hemorrhage volume, operative duration, and intraoperative mechanical ventilation duration are independent predictors of postoperative AA retention.

## Data availability statement

The raw data supporting the conclusions of this article will be made available by the authors, without undue reservation.

## Author contributions

All authors listed have made a substantial, direct, and intellectual contribution to the work and approved it for publication.

## Funding

This study was supported by funding from Special Funding Support for Clinical Medicine Development (ZYLX201708 and DFL20180502) and the Beijing Municipal Science and Technology Commission (Z19110700660000).

## Conflict of interest

The authors declare that the research was conducted in the absence of any commercial or financial relationships that could be construed as a potential conflict of interest.

## Publisher's note

All claims expressed in this article are solely those of the authors and do not necessarily represent those of their affiliated organizations, or those of the publisher, the editors and the reviewers. Any product that may be evaluated in this article, or claim that may be made by its manufacturer, is not guaranteed or endorsed by the publisher.
